# Microbubbles and Nanobubbles with Ultrasound for Systemic Gene Delivery

**DOI:** 10.3390/pharmaceutics12100964

**Published:** 2020-10-14

**Authors:** Yoko Endo-Takahashi, Yoichi Negishi

**Affiliations:** Department of Drug Delivery and Molecular Biopharmaceutics, School of Pharmacy, Tokyo University of Pharmacy and Life Sciences, 1432-1 Horinouchi, Hachioji, Tokyo 192-0392, Japan

**Keywords:** ultrasound-mediated delivery, microbubble, nanobubble, gene delivery, theranostics

## Abstract

The regulation of gene expression is a promising therapeutic approach for many intractable diseases. However, its use in clinical applications requires the efficient delivery of nucleic acids to target tissues, which is a major challenge. Recently, various delivery systems employing physical energy, such as ultrasound, magnetic force, electric force, and light, have been developed. Ultrasound-mediated delivery has particularly attracted interest due to its safety and low costs. Its delivery effects are also enhanced when combined with microbubbles or nanobubbles that entrap an ultrasound contrast gas. Furthermore, ultrasound-mediated nucleic acid delivery could be performed only in ultrasound exposed areas. In this review, we summarize the ultrasound-mediated nucleic acid systemic delivery system, using microbubbles or nanobubbles, and discuss its possibilities as a therapeutic tool.

## 1. Introduction

**Figure 1:**
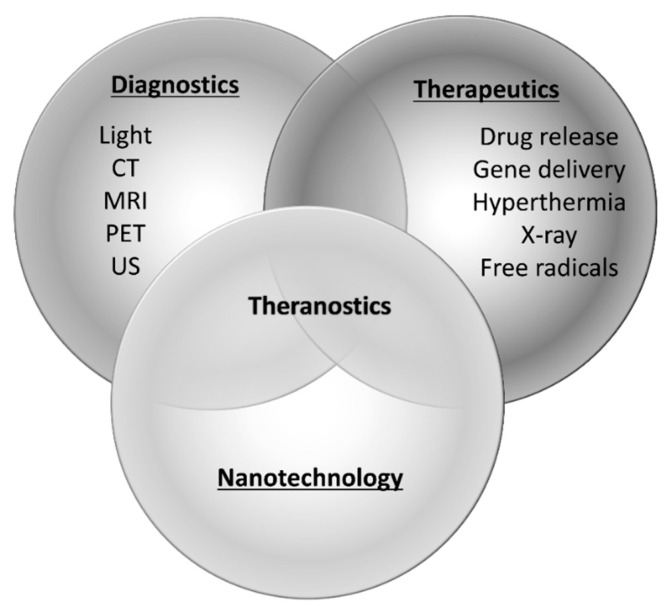
Paradigm of theranostics. CT; computed tomography, MRI; magnetic resonance imaging, PET; positron emission tomography, US; ultrasound.

Gene therapies are expected to be effective therapeutic strategies for intractable diseases such as cancers as well as genetic and inflammatory diseases. In recent years, the number of gene therapy targeted diseases has been increasing, and neurodegenerative and cardiovascular diseases that demonstrate an increased risk due to aging have also been attracting considerable attention as probable targets. For instance, gene therapies for Parkinson’s disease, Alzheimer’s disease, and ischemic disease are actively being studied. However, the safe and efficient delivery of nucleic acids is a major obstacle in their development. In particular, for RNAi-based gene therapies, it is important to avoid degradation by nuclease and their rapid removal from circulation. Various technologies, such as chemical modifications of RNA and nanoparticle-based delivery systems, have been proposed [1,2,3,4,5]. Recently, methods of RNA delivery with external stimuli, including light, ultrasound, electrical fields, and magnetic fields, have been investigated and are expected to improve transfection and therapeutic efficiencies [6,7]. Nanomaterials have been widely used with external stimuli to amplify energy, induce release from carriers, and transfer to target site. These methods show little effect until stimulated with external energy and are thought to be safe for nontarget tissues without stimuli. Furthermore, the development of theranostic (a term for combining therapeutics and diagnostics) nanoparticles has gained attention. Various combinations of nanoparticles with external stimuli are reported to facilitate not only nucleic acid delivery, but also optical imaging, magnetic resonance imaging, nuclear imaging, and computed tomography [8,9,10,11] (Figure 1). Ultrasound imaging is used frequently in clinical settings and valuable for the early detection or follow-up of chronic diseases that increase with age. Furthermore, as it is convenient and non-invasive, ultrasound technology is expected to be a useful theranostic tool. Contrast agents, called microbubbles, are often used in combination with ultrasound to improve the resolution and sensitivity of the imaging. In recent years, nanoscale contrast agents have been developed. Furthermore, micro- or nanobubbles have previously been investigated as site-specific drug or gene delivery tools [12,13,14,15,16,17]. To exploit the combination of ultrasound and bubbles both for diagnosis and therapeutics as a theranostic system, various types of bubbles valuable for systemic administration have been well documented in recent years. This review summarizes systemic gene delivery with microbubbles or nanobubbles in combination with ultrasound.

## 2. Microbubbles and Nanobubbles

**Figure 2:**
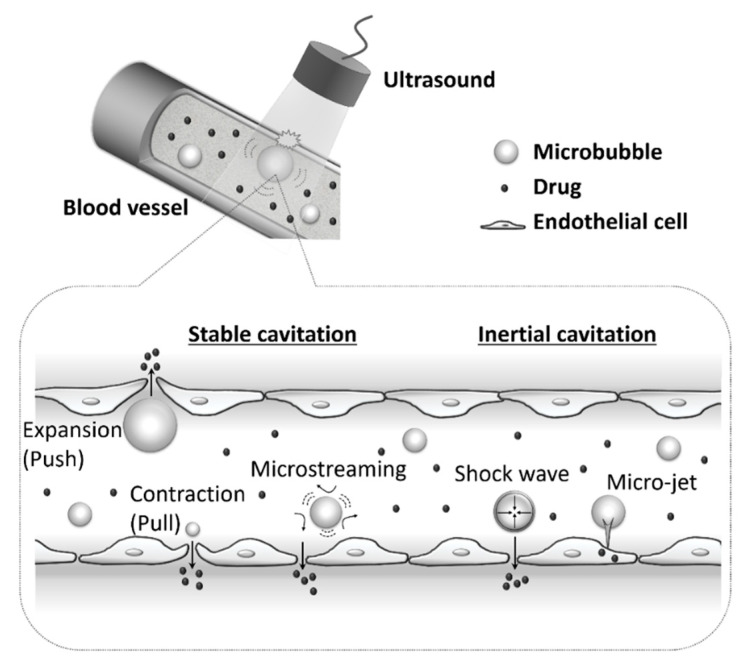
The effects of the combination of ultrasound and microbubbles on cell membrane permeability.

Ultrasound exposure is known to increase cell membrane permeability and facilitate the delivery of drugs or genes into cells [18,19]. A combination of ultrasound exposure and microbubbles further increases cell membrane permeability even when using weak intensity ultrasound, leading to enhanced drug and gene uptake [12,13,14,15,16,17]. Stable oscillations of microbubbles are caused by exposure to low acoustic pressure, a process termed stable cavitation. At higher acoustic pressures, this oscillation may become vigorous and unstable, leading to their collapse and destruction. This phenomenon is called inertial cavitation (Figure 2). The behavior of a microbubble to ultrasound waves depends on the acoustic parameters used [20]. Both stable cavitation and inertial cavitation can be exploited to increase cell membrane permeability. Depending on the bubbles used or the target tissues, it is thought to be necessary to decide ultrasound parameters based on the efficiency of permeability enhancement and the damage to the surrounding cells.

Microbubbles and nanobubbles are usually surrounded by a lipid, protein, or biodegradable polymeric shell structure. The size of the microbubbles generally ranges between 1 and 8 μm and the nanobubbles are submicron sized. Microbubbles are particularly suitable for the molecular imaging of vascular targets, because they are too large to extravasate and accumulate in the perivascular space. However, microbubbles seem to have difficulty in penetrating the deep tissue layers, whereas nanobubbles hold the potential for extensive delivery into tissues through blood vessels. In recent years, nanodroplets have been sought as an alternative to microbubbles. Nanodroplets encapsulate a perfluorocarbon or perfluoropentane core that is stabilized by albumin, lipid, or polymer shells. The cores have low boiling points and the nanodroplets remain in a liquid state at body temperature. Therefore, nanodroplets could pass through the leaky microvasculature and reach the perivascular space, such as a tumor’s interstitial space. Nanodroplets could then be vaporized by ultrasound with high acoustic pressure and converted into microbubbles. It is reported that nanodroplets and ultrasound could be effective tools for imaging, thermal ablation, and drug and gene delivery [21,22,23].

We have previously used liposome technology to developed lipid-based nanobubbles, that are prepared from polyethylene glycol (PEG)-modified liposomes and an echo-contrast gas, perfluoropropane. Liposomes are well studied as useful carriers of drugs, antigens, and genes, and can be easily prepared in a variety of sizes and modified to add specific targeting functions. Furthermore, PEG-liposomes have demonstrated a high biocompatibility and long blood retention time. We considered that these advantages of PEG-liposomes could be also valuable for the preparation of nanobubbles and their applications. First, the liposomes were prepared using neutral lipid and PEG lipid by the reverse-phase evaporation method. Next, echo-contrast gas was entrapped within the liposomes using a bath sonicator to form nanobubbles. We reported that a nanobubble can function not only as an ultrasound contrast agent, but also as a plasmid DNA (pDNA) or small interfering RNA (siRNA) delivery tool with ultrasound exposure, in both in vitro and in vivo [24,25,26,27,28,29]. These in vivo effects were demonstrated following the local injection of a mixed solution with nucleic acids and nanobubbles, followed by transdermal ultrasound exposure to the target tissue. Depending on the target tissues and diseases, the local gene delivery could be highly effective. However, to deliver a gene to deep tissues or vascular endothelial cells, systemic delivery could be more efficacious. Furthermore, to use bubbles in ultrasound imaging as contrast agents, it seems important that bubbles can be adapted for systemic administration. Various nanobubbles have recently been developed and the differences between their functions and those of microbubbles have been investigated [30]. Each bubble has different advantages in the accessible area or the physical effects, and it seems effective to select them, based on the target tissues and objectives. However, in gene delivery via systemic administration, it is important not only that bubbles reach the target tissue and have physical effects on the tissue, but also that the efficiency of gene delivery into target cells is enhanced. Therefore, the additional features mentioned below are needed for microbubbles and nanobubbles to be efficient systemic gene delivery tools.

## 3. Bubbles for Systemic Delivery

**Figure 3:**
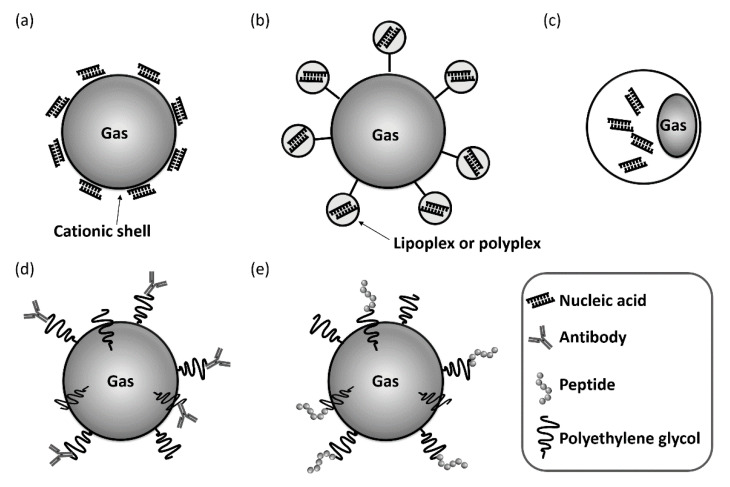
Various types of functionalized bubbles for nucleic acids systemic delivery. (**a**) an electrostatically nucleic acid-loaded bubble (**b**) an complexes-loaded bubble (**c**) a nucleic acid-encapsulated bubble (**d**) an antibody-modified bubble (**e**) a peptide-modified bubble.

After the systemic administration of the mixed solution with free nucleic acids and microbubbles, unprotected nucleic acids are rapidly degraded and removed from circulation. Furthermore, nucleic acids injected intravascularly might not be co-localized with microbubbles, functioning as the driving force of transfection in blood vessels. Based on these reasons, extremely high amounts of nucleic acids are required when administered systemically. In regard to the issue of nucleic acids stability in vivo, it has been reported that nanoparticles of nucleic acids and polymers, such as polyethyleneimine or poly(lactic-*co*-glycolic acid), could increase their transfection efficiencies with ultrasound and microbubbles, via systemic injections [31,32,33]. The formation of nanoparticles with these cationic polymers reduced the risk of the degradation by nuclease and the removal from circulation. Additionally, the positive charge of nanoparticles is expected to increase the interaction with the cell membranes and the uptake into cells. However, the discordance between the intravascular behavior of nucleic acids and bubbles remains unaltered. To address the problem, various types of microbubbles that could load nucleic acids or nanoparticles have been investigated [34] (Figure 3a–c).

### 3.1. Nucleic Acid-Loaded Bubbles

Cationic microbubbles have reportedly been able to load nucleic acids onto their surfaces by electrostatic interaction [35,36,37,38,39]. This results in increased nucleic acid stability and transfection efficiency after intravenous administration. This is a simple method that could be adapted to various negatively charged molecules. We have also developed nucleic acid-loaded nanobubbles using cationic lipids. Nanobubbles containing cationic lipids interacts with pDNA, siRNA, and microRNA (miRNA), with gentle mixing [40,41,42,43]. The loading of nucleic acids onto the surface of nanobubbles containing cationic lipids could increase the stability of nucleic acids in the presence of serum. There was also concern that nucleic acids were degraded by physical force of ultrasound using for transfection. We investigated the damage on siRNA exposed to ultrasound in the same conditions of the transfection. And then, we confirmed that there was no damage to siRNA from ultrasound exposure [40]. It has been also reported that ultrasonic waves do not cause the chains of total RNA to degrade [44]. It might be necessary to take into consideration the effects of ultrasound on nucleic acids depending on the intensity of ultrasound and the kind of molecules.

Previously, we also revealed that cationic nanobubbles were considerably unstable compared to neutral nanobubbles, and that the physical properties, particularly the gas retention ability, were influenced by the liposomal composition. This ability could be improved by altering the unsaturation degree and chain length of fatty acids composing the liposomes. It has been established that short-chain and unsaturated fatty acids increase membrane fluidity of liposomes [45]. Therefore, it was considered that the gas retention ability of nanobubbles was influenced by the lipid membrane fluidity. The use of long-chain and saturated fatty acids as the composition lipids in liposomes could stabilize the nanobubbles. The improvement of the gas retention ability could lead to enhanced abilities in ultrasound imaging and transfection. These results suggested that the lipid composition of nanobubbles was an important factor in diagnostic and therapeutic applications as a theranostics tool. Our nucleic acids-loaded nanobubbles were optimized based on the ultrasound imaging effect, the transfection effect, and the cellular or tissue damage. To evaluate the therapeutic potential of systemically administering these nanobubbles with ultrasound exposure, we have previously used a hindlimb ischemia mouse model and basic fibroblast growth factor (bFGF)-expressing pDNA or miR-126, to promote angiogenesis. The hindlimb ischemia mouse model was used at 10 days post-ligation. After ligation of the femoral artery, the blood flow was repaired in part by spontaneous recovery with the development of collateral circulation, similar to what occurs with chronic diseases. Ultrasound imaging confirmed that our pDNA or miRNA-loaded nanobubbles could reach the ischemic site after intravascular injection. The transfection using the combination of therapeutic ultrasound and these nanobubbles could increase several angiogenic factors, resulting in a significant improvement of the blood flow. Furthermore, the improvement demonstrated in the group with combination therapy were significantly higher than that in the group without ultrasound and that in the group treated with neutral nanobubbles (*p* < 1.0 × 10^−7^ and *p* = 1.8 × 10^−6^). These results suggest that the loading of nucleic acids onto nanobubbles containing cationic lipids is effective for systemic injections. These results also suggested that the nanobubbles could reach their target sites through microscopic blood vessels, via collateral circulation in the ischemic tissues. Therefore, nanobubbles are expected to be useful as diagnosis and delivery tools via systemic administration not only for ischemic tissues, but also for tumors where neovascularization is promoted. Moreover, we considered that the therapeutic effect due to the systemic delivery of miRNA was a significant achievement. It is well established that a single miRNA can regulate the expression of multiple target genes, and it is reported that miRNAs are related to various diseases or aging. Based on this rationale, miRNAs are expected to efficiently regulate disease-related cellular pathways, and considered to be attractive tools and targets for novel therapeutic approaches. We anticipate that the combination of miRNA-loaded nanobubbles with ultrasound could be a useful systemic delivery system, widely applicable across several diseases.

It is also reported that microbubbles can load nucleic acids-liposome complexes (lipoplexes) or nucleic acids-polymer complexes (polyplexes) [46,47,48,49,50,51]. The utilization of lipoplexes or polyplexes could increase the loading volume of nucleic acids onto the bubbles. These strategies which release the complexes from bubbles by the ultrasound exposure could further increase the transfection efficiency compared to methods releasing free nucleic acids. These loading types often use biotinylated PEG to load the complexes onto the microbubbles via avidin-biotin interactions. It was also reported that adeno-associated virus (AAV) could be load onto the microbubbles via avidin-biotin interactions [52]. The electrostatic interactions between the microbubbles and complexes, and the covalent binding of maleimide-PEG on the microbubbles to the thiol group on the complexes, were also employed. The encapsulation of nucleic acids in bubbles has also attempted [22,53,54]. However, the number of reports using nucleic acids-encapsulated bubbles was less than those using cationic bubbles and complexes-loaded bubbles. These results might be due to the difficultly in preparing bubbles that allow for both encapsulation of nucleic acids and gas retention stability in vivo.

### 3.2. Antibody- or Peptide-Modified Bubbles for Targeting Delivery

For the systemic delivery of nucleic acids via ultrasound with microbubbles or nanobubbles, the targeting ability of the bubbles and their loading ability for nucleic acids, are both important. This is due to the accumulation of bubbles by specific ligands for the target site could allow for the enhancement, not only of the imaging effects, but also of the delivery efficiency. For these purposes, antibody-modified bubbles have been developed [55,56,57,58,59] (Figure 3d). For instance, the targeted microbubbles using antibodies against mucosal addressin cell adhesion molecule-1 (MAdCAM-1) or vascular adhesion molecule-1 (VCAM-1) were useful in the ultrasound imaging and gene therapy in crohn’s disease [55]. In case of myocardial infarction, antibodies against matrix metalloproteinase-2 (MMP2) or intercellular adhesion molecule-1 (ICAM-1) could enhance the ability of the microbubbles to increase the effects of imaging and delivery [56,58]. Similarly, it has been reported that the CD105 (endoglin) antibody was available for antiangiogenic tumor therapy, and that microtubule-associated protein-2 (MAP-2) antibody could elevate the therapeutic efficacy in spinal cord injury [57,59]. Thus, antibody-modified bubbles have been well investigated due to their high affinities for target sites. Regarding the development of antibody-modified bubbles, the avidin-biotin interaction has often been adopted as the modification method. However, avidin still remains a challenge of immunogenisity in humans [60]. Therefore, novel methods for the modification of bubbles with antibodies are required for applications in clinical settings. We have recently developed antibody-modified nanobubbles using Fc-binding peptides, derived from proteins A/G [61]. The modification of bubbles with antibody employing Fc-binding peptides could be a simple and feasible method. In fact, we could readily prepare anti-CD146 antibody-modified nanobubbles by our method and demonstrated that the nanobubbles increased the imaging ability of the tumor vessels by modification with the antibody. Although our antibody-modified nanobubbles have not yet been adopted for nucleic acid delivery, they might be a useful targeting delivery and imaging tool.

Peptides that specifically bind to target sites have been well investigated and are often used for the modification of bubbles [62,63,64] (Figure 3e). Regarding the preparation of the targeted bubbles, these studies reported the usability of peptides binding to erythropoietin-producing hepatocellular receptor A2 (EphA2) on tumor cells, to the vascular endothelial growth factor receptor 2 (VEGFR2) of tumoral endothelium, or to integrin αvβ3 of endothelial cells. We have also successfully developed targeted nanobubbles modified with end of PEG chains with AG73 peptides (AG73) [65,66]. AG73 is derived from the globular domain of the laminin α1 chain and considered a ligand for syndecans, a major heparan sulfate-containing transmembrane proteoglycans [67,68]. Furthermore, syndecan-2 is highly expressed in neovascular vessels [69,70], and AG73-modified nanobubbles showed specific attachment, gene delivery, and ultrasound imaging abilities for tumor neovessels, both in vitro and in vivo [65,66]. We have previously prepared Angiopep-2 peptide (Ang2)-modified nanobubbles in a similar manner to the AG73-modified nanobubbles [71]. Notably, Ang2 is considered for brain-targeted delivery of drugs, genes, and peptides [72,73,74,75,76]. It is reported that Ang2 bind to bEnd.3 cells via low-density lipoprotein receptor-related protein-1 (LRP1) [77,78]. The bEnd.3 cell-line has been used as a model for the blood-brain barrier (BBB), owing to their rapid growth and maintenance of BBB characteristics over repeated passages. Ang2-modified nanobubbles could bind to bEnd.3 cells via LRP1. Furthermore, the binding ability could be enhanced by combining peptide binding long PEG chain and short PEG chain without peptide. Reportedly, it has been demonstrated that the modification of liposomes with long and short PEG chains alters the conformation of the PEG chains and results in an increase in the fixed aqueous layer thickness [79]. It was suggested that the long PEG chains were extended by the presence of the short PEG chains, resulting in Ang2 that could easily attach to the LRP1 expressed on the cell surfaces. Ang2-moodified nanobubbles also could enhance the accumulation and the brightness of the ultrasound images in the brain after systemic administration. In addition, the gene delivery effects in the brain tissue by the ultrasound and Ang2-modified cationic nanobubbles loading pDNA were significantly higher compared to those in the group treated by the control peptide-modified nanobubbles.

Proteins and saccharides, such as transferrin and mannose, were also used for the modification of the bubbles as a ligand [80,81]. These reports suggested that targeted bubbles modified with various ligands could be useful tools not only for ultrasound imaging, but also for nucleic acid delivery to target sites via systemic administration.

## 4. Various Ultrasound Devices and Ultrasound Delivery in Clinical Use

**Figure 4:**
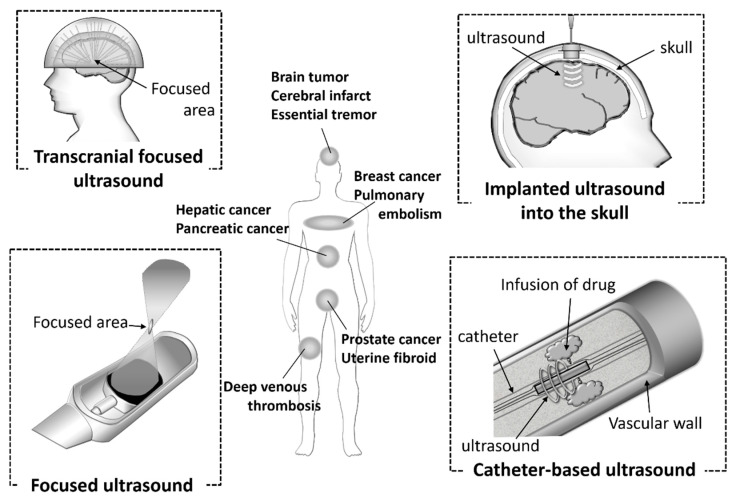
Ultrasound devices used in clinical settings.

Ultrasound technology is essential for the diagnosis of various diseases due to its noninvasiveness, small device size, simple and real-time operations, and low costs. In addition, the technology is also used in clinical settings as therapeutic equipment for calculi, tumors, bone fractures, and Parkinson disease. It has attracted attention as a potential energy for use with theranostic systems. In recent years, ultrasonic devices have being actively developed (Figure 4). Catheter-based ultrasound and MRI-guided focused ultrasound have already been utilized in clinical settings. Computer-controlled ultrasound systems can provide precise exposure to the target sites. These devices are used for thrombolysis in the treatment of diseases, such as cerebral infarct, pulmonary embolism, and deep venous thrombosis, and the ablation of hepatic cancer, prostate cancer, breast cancer, and uterine fibroids. Furthermore, it has been reported that the combination of ultrasound and microbubbles could enhance gemcitabine treatment of inoperable pancreatic cancer in clinical trials [82].

The approval of a transcranial focused ultrasound system has also been obtained for the treatment of essential tremors. Ultrasound devices have also been used for sonodynamic therapy (SDT). SDT is a cancer therapy in which the sensitizer accumulated in the tumor cells is activated by ultrasound and produces free radicals. SDT can provide deep penetration of the target cancer cells compared with photodynamic therapy (PDT), therefore the combination of sonodynamic and photodynamic therapy (SPDT) can been adopted, depending on the area of the tumor. Ultrasound diagnosis and treatment are already widespread and actively developed. The functionalized microbubbles or nanobubbles discussed in this review have the potential to enhance these effects.

Recently, BBB openings using microbubbles and ultrasound have been focused on as a novel therapeutic strategy for central nervous system (CNS) diseases. The BBB limits the delivery of systemically administered drugs to the brain, so as to protect it from exposure to potentially damaging substances. Therefore, the BBB has been main obstacle to delivering valuable therapies for CNS diseases. It has been reported that using focused ultrasound-mediated microbubbles enabled a transient BBB opening in a localized brain region [83,84]. Therapeutic ultrasound can cause the oscillation and collapse of microbubbles. Stable and inertial cavitation enables microbubbles not only to cause transient disruptions in the cell membranes, but also to increase the BBB permeability. In fact, the combination of our nanobubbles with focused ultrasound could also serve to increase the BBB permeability [85]. The BBB permeability was assessed using Evans blue dye. It is well known that Evans blue dye stably binds to serum albumin. Furthermore, the extravasation of Evans blue dye indicates albumin extravasation. The opening of the BBB with nanobubbles and focused ultrasound was confirmed by the extravasation of Evans blue dye preinjected prior to ultrasound exposure. We demonstrated that the effect of BBB opening was transient. Additionally, the BBB permeability and tissue damage were influenced by the ultrasound intensity, exposure time, and molecular size. Furthermore, it was also reported that phosphorodiamidate morpholino oligomer (PMO), which is one of the chemically modified nucleic acids, or pDNA could be delivered to ultrasound exposed cerebral hemispheres. We considered that a similar situation occurred when pDNA was transfected to the brain using the combination of ultrasound and Ang2-modified cationic nanobubbles mentioned above. Many research groups have reported the effects of BBB opening and the potential applications of gene therapy using pDNA, siRNA, or AAV [86,87,88,89,90,91,92,93]. In a clinical setting, the state-of-the-art MRI-guided focused low-intensity ultrasound was used for a patient with a malignant brain tumor to deliver chemotherapeutic agents in 2015 [94]. In the next year, another group also reported that the chemotherapeutic agent was delivered to the glioblastoma using a pulsed ultrasound device implanted into the skull of the patient [95]. In both therapeutic strategies, microbubbles were used with transient BBB openings. CNS diseases are often intractable, and tissue damage to the not therapeutic area can easily lead to disabilities. Therefore, ultrasound-mediated diagnosis and therapies using computer-controlled precision devices and functionalized bubbles might have significant benefits.

## 5. Concluding Remarks and Future Perspectives

The mechanisms of aging and the onset of many diseases have been identified at the genetic level. Furthermore, the elucidation of gene regulatory networks and the development of technologies for nucleic acid synthesis are also progressing rapidly. In these situations, novel therapies with nucleic acids have become increasingly important. The delivery of nucleic acids by a combination of ultrasound and micro- or nano-bubbles, has been shown to be effective in the treatment of diseases such as cardiovascular disease, CNS disease, and tumors. Low power ultrasound technology, which is used for imaging, is well known to be noninvasive and can be used repeatedly. Whereas, high power ultrasound already used clinically, for instance in the ablation of cancer, could cause undesirable bioeffects due to its high intensity [96]. Therefore, high power therapeutic ultrasound is used, paying careful attention to the safety of patients. Unfortunately, it is not obvious the bioeffects by ultrasound for micro- or nanobubbles-based gene therapies. For the realization of clinical application, more detailed information about safety is required, and it is essential to establish appropriate ultrasound conditions which can also be used repeatedly. Owing to the possibility of repeated use and device miniaturization, it is particularly advantageous in elderly patients, particularly for in home care which will continue growing in the near future. However, the development of bubbles which is useful both for diagnosis and therapy is not sufficient, although various ultrasound devices are developed remarkably and widely spread in clinical settings. As the development of useful bubble formulations progresses, the combination of bubbles and ultrasound is highly valuable for the assembly of a theranostic system in the diagnosis and therapy of diseases that increase in risk due to aging.
